# How Trust in Information Sources Influences Preventative Measures Compliance during the COVID-19 Pandemic

**DOI:** 10.3390/ijerph18115867

**Published:** 2021-05-30

**Authors:** Sherry A. Maykrantz, Tao Gong, Ashley V. Petrolino, Brandye D. Nobiling, Jeffery D. Houghton

**Affiliations:** 1 School of Health Sciences, Salisbury University, Salisbury, MD 21801, USA; samaykrantz@salisbury.edu (S.A.M.); avpetrolino@salisbury.edu (A.V.P.); bdnobiling@salisbury.edu (B.D.N.); 2 Department of Social Sciences, University of Maryland Eastern Shore, Princess Anne, MD 21853, USA; tgong@umes.edu; 3 Department of Management, West Virginia University, Morgantown, WV 26506, USA

**Keywords:** COVID-19 pandemic, trust, information sources, self-efficacy, perceived susceptibility

## Abstract

This paper explores how trust in formal information sources (government and media) and informal information sources (interpersonal) about COVID-19 influences compliance with preventive measures. This cross-sectional study uses convenience sampling of 478 adult participants. Data analyses using structural equation modeling with multigroup comparisons examine hypothesized relationships between trust in information sources and preventative behaviors and social distancing. Results suggest that understanding of COVID-19 causes is related to trust in formal information sources, but not to trust in informal information. Self-efficacy for prevention is related to trust in informal information sources, but not to trust in formal information sources. Worry about contracting COVID-19 is related to trust in formal information sources, but not to informal ones. Engaging in preventive measures is linked to both self-efficacy for prevention and worry, while social distancing was related only to worry. These findings have important implications for public health policy guidelines centered on clear and truthful media messages. The findings also facilitate comparative analyses of reactions to information sources across a decade of evolving attitudes toward media and government, between two cultures (Hong Kong vs. the USA), and between two different global pandemics.

## 1. Introduction

In March 2020, the United States of America (USA) declared a national emergency, put international travel restrictions into place, suggested social distancing at all levels of society, and advised anyone who felt sick and/or households with anyone testing positive to stay home. In addition, older Americans and those with underlying health conditions were warned to take extra precautions to protect their health. In April of 2020, one month after COVID-19 was declared a global pandemic, [1] the CDC published guidelines that included face coverings, hand washing, and using hand sanitizer [2]. Although the federal government of the USA, in collaboration with the CDC and other health experts, announced these suggestions, support varied among state and local leaders and across traditional media outlets [3]. This resulted in mixed and conflicting messaging that undermined public trust of various information sources.

Consequently, exploring trust perceptions of various sources of health information and associated perceptions of infection risk and self-efficacy for prevention may provide insight into what factors influence self-protective decision-making and behaviors. For example, the perceived risk of SARS and self-efficacy for SARS prevention varied based on the country and region in which a person lived at the time for people in some European countries and certain regions of China [4]. Such research highlights the need for additional efforts to determine the individual influences related to risk-perception discrepancies. Indeed, Leppin and Aro [5] suggest that public health planning in pandemic situations “will be highly dependent on sound theory-based research on how people perceive the risks involved in such an event” (p. 7).

The purpose of the current study is to examine how trust in formal (i.e., government and media) and informal (i.e., family and friends) information sources about COVID-19 influences compliance in practicing preventive measures in the specific context of the USA. Preventative measures consist of recommendations to wear a mask in public, maintain social distancing of at least six feet, wash hands regularly, and use hand sanitizer. To facilitate this aim, we adapt the conceptual model and measures of Liao et al. [6] to the current context of the COVID-19 pandemic and the culture of the USA. Applying this model in a different time and context provides an opportunity to make comparisons of reactions to information sources across a decade of evolving attitudes toward media and government, between two cultures (Hong Kong vs. the USA), and between two considerably different global pandemics (H1N1 vs. COVID-19).

The COVID-19 pandemic has underscored a long-time theme within the public health field regarding influences on preventative measures. Public health professionals have grappled with the duality and necessity of informal and formal information routes to influence preventative measures ever since John Snow linked a cholera outbreak to the Broad Street pump in London during the 19th century [7]. Current principles of risk communication are based on information gleaned from research during past public health emergencies [8], which are showcased in the management, psychology, and communication sciences. These studies have shed light on what influences trust in formal and informal public health information sources and therefore an individual’s compliance with preventative measures during a crisis. Henrich and Holmes [9] found that the majority of participants in their study perceive family doctors and websites such as the CDC and WHO as the most trusted sources of information in a pandemic. Similarly, Bradley et al. [10] found a positive link between trust in formal information sources and preventative/protective behaviors that can reduce the risk of transmission of COVID-19. In addition, recent studies have found that political ideology, and public health considerations, influence compliance to COVID 19 preventative measures [11,12]. Likewise, researchers [13,14] found differences in political party and gender when seeking informational sources about COVID-19. Finally, Verma et al. [15] reported that the participants in their study showed good knowledge of primary and secondary COVID-19 symptoms, infection spread mechanisms, and preventive measures including masks, sanitizers, and hand washing, while referencing the World Health Organization website as their primary source of information.

## 2. Theoretical Frameworks

The health belief model (HBM) and the theory of planned behavior (TPB) were used as theoretical foundations for examining the role of trust in information sources in predicting preventive measures and social distancing. Both theories have been used to predict behaviors in public health research for decades and serve well for the current study. The HBM was developed to help explain why individuals neglect to participate in health screenings and has since become established as perhaps the most widely used contemporary health behavior theory [16]. The HBM suggests that six constructs (perceived susceptibility, perceived severity, perceived barriers, perceived benefits, self-efficacy, and cues to action) are beneficial in forecasting individual [17]. The current study applies two of these six constructs, investigating both perceived personal susceptibility (how vulnerable does one believe they are to contracting COVID-19) and self-efficacy of COVID-19 prevention. Callow et al. [18] note that “people are more likely to adopt a health behavior (i.e., stay at home) if they believe they are at high risk of being infected (perceived susceptibility)” (p. 2). Furthermore, the role of self-efficacy, defined as belief in one’s ability to take action, in facilitating changes in health behaviors is well established in the literature [19].

**Figure 1:**
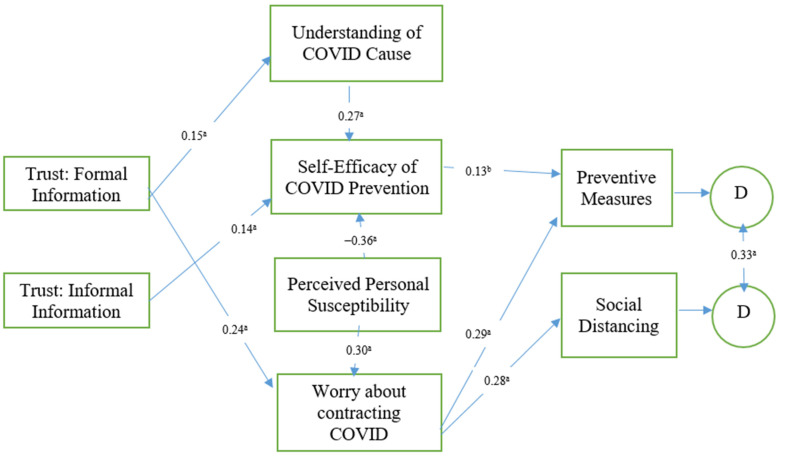
The standardized coefficients for the hypothesized structural equation model. ^a^ *p* < 0.001. Nonsignificant paths excluded for clarity.

The TPB posits that attitude, subjective norms, and behavioral intentions, coupled with perceived behavioral control toward the behavior, serve as valuable predictors of whether or not one chooses to engage in a particular behavior [20]. The current study seeks to advance the understanding of how the TPB can predict preventive measures (i.e., wearing face covering, washing hands, etc.) and social distancing compliance. Specifically, in our hypothesized model (shown in Figure 1), the extent to which people trust formal and informal sources of information shapes their beliefs and attitudes concerning COVID-19 causes, including their self-efficacy for prevention, their perceived susceptibility, and their worry about contracting COVID-19. These beliefs and attitudes subsequently shape behavioral intentions and actual behaviors relating to preventive measures and social distancing.

Recently, Callow et al. [18] utilized both HBM and the TPB as theoretical frame for their study, which found support for a conceptual model in which attitudes toward social distancing predict intentions to socially isolate. Similarly, Qazi et al. [21] used the TPB to explore a hypothesized model in which formal and informal sources of information predict perceived understanding, which in turn predicts social distancing behaviors. The current study goes beyond these findings by exploring not only perceived understanding of COVID-19 but also self-efficacy, perceived susceptibility, and worry as additional hypothesized mediators, and not only social distancing but also other preventive measures as additional hypothesized behavioral outcomes. Consequently, our study provides a richer explanation of how trust in formal and informal information sources affect health related behaviors such as compliance to preventive measures. Our study advances public health research and makes an important contribution to theory, policy and practice aimed at improving population health.

## 3. Materials and Methods

### 3.1. Data Collection Procedures

Upon approval from the institutional review board (IRB) at our affiliated university, subjects were recruited in two ways: (1) The electronic link for this survey was posted on several social media platforms, forwarded through email contact listings, and sent out through regional listservs and newsletters; (2) through the networks (e.g., relatives, acquaintances, and social media) of students in our classes, who were encouraged to share the survey link with individuals over the age of 18. Meta-analytic analyses suggest that student-recruited sample demographics do not differ substantively from non-student recruited samples, with very similar observed correlations and only somewhat smaller effect sizes [22]. All potential participants were given the option to choose to not participate. All data were collected from respondents in the USA during the first week in May 2020, resulting in 531 responses. We used listwise deletion to eliminate cases with missing data and cases that did not meet the minimum inclusion criteria, resulting in a final sample size of 478. 

### 3.2. Participants

In total, 478 respondents participated in this study, with 63% between the ages of 18 and 37. Gender representation was 26% male and 74% female. Race was broken down as 78% White and 17% Black, 5%. Political party was 43% democrats, 28% republicans, 20% independents, with 7% no political party, and 2% other. A total of 50% of participants had a bachelor’s degree or higher. 

### 3.3. Instruments

We adapted the instruments used by Liao et al. [6] in measuring the variables analyzed in this study. Trust in formal and informal information sources was measured using a 5-point Likert scale by asking: (1) I am persuaded by what I read in the paper or online about COVID-19. (2) Media reports about COVID-19 can be trusted. (3) I trust what the government says about COVID-19. (4) The best source of information about COVID-19 is to watch others and listen to what they say. (5) I tend to believe what my friends, colleagues, or neighbors say about COVID-19 rather than the papers, news, or social media. Responses ranged from “strongly disagree” to “strongly agree”. Understanding of COVID-19 transmission and efficacy beliefs were assessed with single items (i.e., I understand how people get infected with COVID-19 and I am confident that I can prevent myself from catching COVID-19) using a 5-point Likert scale with responses from “strongly disagree” to strongly agree”. 

Perceived susceptibility was measured using a 5-point Likert scale applied to the following questions: (1) how likely do you think it is that you will contract COVID-19 over the next 1 month? (2) how likely is it that you will contract COVID-19 compared with other people of your social group? Both questions generated responses ranging from, “strongly disagree” to “strongly agree.” Worry included one item (i.e., in the past one week, have you ever worried about catching COVID-19?) using a 5-point Likert scale with responses ranging from “never” to extremely”. Preventive actions used a 4-point Likert scale to measure six items (e.g., in the past 30 days I have worn a face mask/covering when outside). Finally, social distancing behaviors were measured with four items (e.g., I avoid going out (grocery store, drug store, and convenience stores) due to COVID-19) using a “Yes”–“No” response. Reliability coefficients for the instruments are shown in Table 1.

### 3.4. Statistical Analysis

We conducted descriptive statistical analysis, bivariate correlational analysis, and path analysis for the relationships in the hypothesized model (see Figure 1). Prior to testing the structural model, we analyzed the internal validity of each instrument for measurement errors and found all of the instruments were reliable and above the minimum threshold of 0.5 [23]. As a result, we conducted path analysis in testing the relationships in the hypothesized model. 

## 4. Results

Table 1 reports descriptive statistics and Cronbach alphas for the instruments. We found prevention and social distancing measures were significantly correlated with formal information sources (media and government) and with worry about contracting COVID-19.

### 4.1. Hypothesized Model

To analyze the relationships in the hypothesized structural model, a path analysis was conducted. After inspecting the fit indices of the hypothesized model, we found it fits the data well, χ^2^ (df = 18) = 18.559, *p* = 419, RMSEA = 006 less than (≤08) and CI_95_ [0.000, 0.030], NFI = 977 greater than 95, CFI = 999 greater than 95 (≥95), and SRMR = 019 less than 06 (≤06). As hypothesized, trust in formal sources of information (media and government) is significantly related with understanding of COVID-19 cause, β = 15 and *p* < 0.001 and worry about contracting COVID-19, β = 24 and *p* < 0.001, whereas trust in informal information sources (interpersonal) was significantly related with self-efficacy of COVID prevention, β = 14 and *p* < 0.001. Additionally, as hypothesized, worry about contracting COVID-19 was significantly related with prevention measures, β = 29 and *p* < 0.001, and social distancing measures, β = 28 and *p* < 0.001. Further and as expected, self-efficacy of COVID-19 prevention was significantly related with prevention measures, β = 13 and *p* < 0.01. Additionally, understanding of COVID-19 cause was significantly related with efficacy of COVID-19 prevention, β = 27 and *p* < 0.001, while perceived personal susceptibility had a significantly positive relationship with self-efficacy of COVID-19 prevention, β = 36 and *p* < 0.001, and a significantly negative relationship with worry about contracting COVID-19, β = −0.30 and *p* < 0.001, respectively. Figure 1 shows the standardized coefficients for the hypothesized structural equation model.

### 4.2. Gender Differences

**Figure 2:**
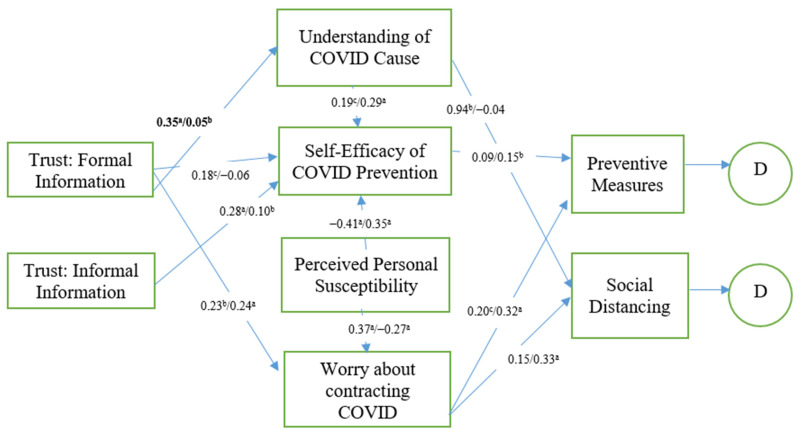
Multigroup comparison of the standardized coefficients between males and females. ^a^ *p* < 0.001, ^b^ *p* < 0.01, and ^c^ *p* < 0.05. Nonsignificant paths excluded for clarity. Significant differences between males and female are shown in bold.

Next, a multigroup analysis was conducted to determine if there is a difference between males and females for the relationships in the hypothesized model. Figure 2 reports the standardized coefficients and their significance levels. The path estimates for male respondents are shown first in the figure. Among the relationships in the model, only one pathway/relationship, trust in formal information (media and government) and understanding of the COVID-19 cause, were significantly different between male and female participants, with the standardized coefficient for male participants, β = 35 and *p* < 0.00, being significantly stronger than that of female participants, β = 05 and *p* < 0.01. A bootstrapping bias-corrected percentile method was used to estimate a 95% confidence interval for the difference in the standardized estimates between males and females. The difference in the estimates was 0.104 and CI_95_ = [0.057, 171], which was significant at *p* < 0.01. It is interesting to note that there were two relationships/pathways only significant for males. The relationship between trust in formal information (media and government) and self-efficacy of COVID-19 was positive and significant at 0.05 level, while the relationship between the understanding of the COVID-19 cause and social distancing was positive and significant at 0.01 level. Similarly, two relationships/pathways were significant only for females. The relationship between self-efficacy of COVID-19 prevention and preventive measures was positive and significant at 0.01 level, whereas the relationship between worry about contracting COVID-19 and social distancing was positive and significant at the 0.001 level.

### 4.3. Cross-Cultural Differences

Examining the results of the current study with those of Laio et al. [6] allows for some interesting comparisons across time (2010 vs. 2020), cultures (Hong Kong vs. the USA), and pandemics (H1N1 vs. COVID-19). Path estimates from both samples are summarized in Table 2. Laio et al. [6] reported significant relationships between trust in informal (interpersonal) information and both perceived personal susceptibility and worry about contracting, whereas our current study did not. Further, the current study found a significant relationship between trust in formal information sources and worry about contracting, while Laio et al. [6] did not. Finally, Laio et al. [6] reported a significant relationship between prevention self-efficacy and social distancing, whereas our current analysis did not. 

## 5. Discussion

This study examined a hypothesized model in which the effects of the predictor variables trust in formal and informal information sources are mediated through beliefs and attitudes (understanding the cause, prevention self-efficacy, perceived susceptibility, and worry about contracting) to influence behaviors relating to preventative measures of mask-wearing and hand washing along with social distancing. Our findings suggest that people’s understanding of COVID-19 causes is influenced by trust in formal information sources, but not by trust in informal information. Conversely, prevention self-efficacy is influenced by trust in informal information sources and not by trust in formal information sources. Our results also show that worrying about contracting COVID-19 is related to trust in formal information sources but not informal ones. Finally, we found that engaging in preventive measures behavior (i.e., mask-wearing and handwashing) was predicted by both self-efficacy for prevention and worry, while social distancing was influenced only by worry about contracting COVID-19.

Comparing outcomes for males and females, the only significant difference in coefficients was for the path between trust in formal information sources and understanding of COVID-19 causes, suggesting that men or more likely than women to use formal information from media and government sources to inform their understanding of what causes COVID-19. However, certain path estimates were only significant for either males or females. For example, men significantly relied upon formal information sources in shaping their prevention self-efficacy beliefs whereas women did not. Similarly, men’s social distancing behaviors were significantly influenced by their understanding of the causes of COVID-19, while women’s understanding was not. In contrast, women’s preventative measures behaviors of mask-wearing and handwashing were significantly shaped by their self-efficacy perceptions, while men’s were not. Finally, women’s social distancing behaviors were significantly related to their worry about contracting COVID-19, whereas men’s were not. Taken together, these findings suggest that men tend to rely on rational decision-making processes based on their trust in formal information sources provided by media and government and their understanding of the causes of COVID-19 for shaping their health-protective behaviors. Women, on the other hand, tend to rely more on emotive decision-making based on their concerns about contracting COVID-19 in shaping their health-protective behaviors. Gender differences in personality could help account for our differential findings. For example, meta-analytic data across 26 different cultures found that women reported being higher in the personality traits of neuroticism, agreeableness, warmth, and openness to feelings, while men reported themselves as being higher in assertiveness and openness to ideas [24].

Although direct comparisons of the findings of the current study and those of Laio et al. [6] are problematic given the substantial cultural, temporal, and contextual differences characterizing each data collection, we nevertheless offer some speculative interpretations and explanations for the differences summarized in Table 2. First, the significant relationships between trust in informal information and both perceived personal susceptibility and worry about contracting COVID-19 as reported for the Hong Kong sample could be explained by the idea that, in the more collectivist culture of Hong Kong, people trust and rely more on interpersonal information to form beliefs and attitudes than in the individualistic culture of the USA. Alternatively, the more stringent nature of the lockdowns and quarantines during the COVID-19 pandemic may have curtailed interpersonal information sources more than during the H1N1 pandemic. Second, the significant relationship between trust in formal information sources and worry about contracting COVID-19 in the current USA sample may be due to the greater magnitude and intensity of media coverage and governmental communications during the COVID-19 pandemic relative to the H1N1. Finally, the significant relationship between prevention self-efficacy and social distancing reported for the Hong Kong sample could also relate to the collectivist/individualistic distinction, particularly personal proxemics norms [25,26]. People in the USA may already be practicing greater social distancing than people in Hong Kong and therefore the effects of prevention self-efficacy beliefs were more significantly related to mask-wearing and handwashing behaviors in the current sample.

The results of this study offer several implications for public health policy and practice. First, our study revealed the importance of trust in formal (government and media) informational sources, and the role it plays in peoples understanding of COVID-19. Therefore, policy makers should be cognizant of the information being put out on media sources. Public health policies and guidelines should be put in place for clear and truthful media messages. Second, worry about contracting COVID-19 was also related to formal informational sources. This calls for attention toward what and how government officials (federal, state, and local) provide information to the public. Third, public health experts should work with policy makers to articulate the importance of using gain-framed massages that appeal to both genders (e.g., I feel confident about my ability to prevent COVID -19 simply by talking to my friends about hand washing, face masks, etc.).

Our study is subject to certain limitations. First, the cross-sectional nature of our data limits generalizability. Second, some of our survey instruments demonstrated marginal reliability. However, all estimates were above 5, a common threshold of acceptability [23]. Third, political party affiliation was skewed (43% democrat vs. 28% republican), which further limits generalizability. Fourth, our study collected data from various states throughout the USA, a potential limitation because each state and region were experiencing different circumstances. Finally, as noted above, direct comparisons between the USA sample data collected for the current study and the Hong Kong sample data collected by Laio et al. [6] are problematic given the substantial differences in the two populations and data collection contexts. Consequently, the interpretations regarding the cross-cultural differences advanced above should be viewed with a degree of caution. Nevertheless, these comparisons may offer some basic insights into possible differences in reactions to information sources across time, culture, and context.

## 6. Conclusions

In summary, this study examines a hypothesized model of how trust in formal and informal information sources about COVID-19 in the USA influences compliance with preventive measures. Our findings suggest that trust in formal information sources influences understanding of COVID-19 causes, while trust in informal information sources shapes self-efficacy for prevention. Trust in formal information sources is related to worry about contracting COVID-19, while self-efficacy is linked to preventative measures and worry is related to both preventive measures and social distancing.

Future studies should expand the model examined here by investigating additional attitudinal mediators and health protective behaviors. Likewise, future studies could explore other theoretical dimensions of the HBM and/or TPB for an ever-richer theoretical understanding of how sources of information affect health behaviors in a pandemic. Future research could also focus on other cultures of interest. For example, people experiencing the COVID-19 pandemic in European countries could react differently to information sources than people in the USA or Hong Kong. Ideally, future studies could collect data across multiple cultures simultaneously to avoid some of the limitations of the cross-cultural comparisons made here. Finally, future research could examine the role of demographic factors such as political affiliation, age, education, and race in public compliance with preventive measures. For example, the age of the participants in our study would almost certainly influence health protective behaviors given differences and perceived differences in risk factors across generations. In such studies, multiple group comparisons would be particularly valuable in developing targeted health communication campaigns and public health messaging for specific audiences, which is the nucleus of effective public health communication.

## Figures and Tables

**Table 1:** Mean, standard deviations, and zero-order correlation coefficients.

	M	STD	1	2	3	4	5	6	7	8
1. Formal	8.72	2.51	(0.62)							
2. Informal	4.37	1.67	0.08	(0.50)						
3. Understand	4.41	0.80	0.14 **	0.08						
4. Efficacy	3.70	1.07	0.04	0.13 **	0.27 ***					
5. Susceptible	7.12	1.28	−0.00	0.03	0.04	0.37 ***	(0.75)			
6. Worry	3.48	1.07	0.24 ***	−0.01	0.03	−0.15 ***	−0.30 ***			
7. Preventive	19.00	2.85	0.14 **	−0.02	0.08	0.10 *	−0.02	0.26 ***	(0.66)	
8. Distancing	18.89	2.32	−0.19 ***	−0.00	−0.10 *	−0.02	0.01	−0.28 ***	−0.42 ***	(0.51)

*N* = 478. *, **, and ***: Significant at the 0.05, 0.01, and 0.001 levels (2-tailed). Reliability coefficients are shown in diagonal.

**Table 2:** Comparison of path estimates across USA and Hong Kong samples. ^a^ *p* < 0.001, ^b^ *p* < 0.01, and ^c^ *p* < 0.05. Nonsignificant paths excluded for clarity.

Path	USA	Hong Kong
Trust Formal→Understanding	0.15 ^a^	0.36 ^a^
Trust Formal→Self-Efficacy		0.25 ^a^
Trust Formal→Susceptibility		
Trust Formal→Worry	0.24 ^a^	
Trust Informal→Understanding		
Trust Informal→Self-Efficacy	0.14 ^a^	
Trust Informal→Susceptibility		−0.21 ^a^
Trust Informal→Worry		0.16 ^b^
Understanding→Self-Efficacy	0.27 ^a^	
Susceptibility→Self-Efficacy	−0.36 ^a^	−0.42 ^a^
Susceptibility→Worry	0.30 ^a^	0.44 ^a^
Understanding→Preventative		0.19 ^a^
Understanding→Distancing		
Self-Efficacy→Preventative	0.13 ^b^	0.23 ^a^
Self-Efficacy→Distancing		0.13^c^
Susceptibility→Preventative		
Susceptibility→Distancing		
Worry→Preventative	0.29 ^a^	0.13 ^c^
Worry→Distancing	0.28 ^a^	0.36 ^a^

## Data Availability

Data available on request due to privacy restrictions. The data presented in this study are available on request from the corresponding author. The data are not publicly available due to authors preference.
